# Systemic dysregulation of the gut microenvironment plays a pivotal role in the onset and progression of inflammatory bowel disease

**DOI:** 10.3389/fimmu.2025.1661386

**Published:** 2025-09-25

**Authors:** Ruilong Kou, Yonggang Guo, Zhiwei Qin, Xiaochen Xu, Yihao Liu, Wenqin Wei, Yu Chen, Zhiyuan Jian, Bin Lan

**Affiliations:** ^1^ Department of Gastrointestinal Surgery 2 Section, The First Affiliated Hospital, Fujian Medical University, Fuzhou, China; ^2^ Department of Gastrointestinal Surgery, National Regional Medical Center, Binhai Campus of the First Affiliated Hospital, Fujian Medical University, Fuzhou, China; ^3^ Department of Gastrointestinal Surgery, Affiliated Hospital of Guilin Medical University, Guilin, China; ^4^ Pingdingshan University, Pingdingshan, Henan, China

**Keywords:** inflammatory bowel disease (IBD), gut microenvironment, gut microbiota, metabolites, intestinal epithelial barrier, immune system

## Abstract

Inflammatory bowel disease (IBD) represents a multifaceted, chronic inflammatory condition affecting the gastrointestinal tract, with its underlying pathophysiological mechanisms not yet fully elucidated. Recent research has underscored the pivotal role of the gut microenvironment, a complex ecological system, in the pathogenesis of IBD. This review systematically examines the interactions between gut microenvironment components and their roles in the pathogenesis of IBD. It is now understood that gut dysbiosis results in a decrease in beneficial microbiota, such as *Faecalibacterium* and *Roseburia*, along with an increase in pathogenic bacteria, including Adherent-invasive *Escherichia coli (AIEC)*. This microbial imbalance results in a reduction in the production of beneficial metabolites, such as short-chain fatty acids, and the accumulation of detrimental metabolites, thereby directly disrupting the gut microbiome. The resultant gut dysbiosis leads to dysfunction in intestinal stem cells (ISCs) and a reduction in the expression of tight junction (TJ) proteins, thereby further compromising the integrity of the intestinal epithelial barrier. This dysfunction allows microorganisms and harmful metabolites to penetrate the barrier, reaching the submucosal layer, where they activate both innate and adaptive immune responses, thereby initiating a complex immune cascade. Over time, this process leads to a self-sustaining inflammatory cycle that culminates in chronic IBD and potentially contributes to the development of metabolic disorders. This paper examines this cycle, elucidating the interactions among gut microbiota dysbiosis, metabolite alterations, barrier dysfunction, and immune activation that drive the pathogenesis of IBD, while also critically assessing the limitations of current therapeutic strategies. Based on our understanding of the overarching dysregulation of the gut microenvironment, we propose a paradigm shift in IBD from “controlling inflammation” to “restoring intestinal homeostasis”, and from “single therapy” to “comprehensive intervention”. This integrated approach encompasses microbiome remodeling, metabolite intervention, reconstruction of the immune microenvironment, and repair of barrier function. Such a multidimensional and integrated therapeutic strategy promises to effectively disrupt the pathological feedback loop, restore gut homeostasis, and offer novel theoretical and clinical insights for the precise treatment of IBD and its progression.

## 1 Introduction

Inflammatory Bowel Disease (IBD) is a complex, chronic gastrointestinal disorder influenced by multiple factors and primarily characterized by persistent intestinal inflammation. Although the pathogenesis of IBD remains incompletely understood, current research suggests that its core pathological processes involve an interplay among dysregulated microorganisms and metabolites, intestinal barrier dysfunction, immune activation, and the progression of chronic inflammation (1–4).

The gut microenvironment constitutes a complex ecosystem comprising microorganisms and their metabolites, the intestinal epithelial barrier, the immune system and the circulatory system, all of which are essential for maintaining human health. Its primary functions include regulating metabolism, preserving barrier integrity, promoting immune balance, and protecting the body’s intrinsic systems from external harmful factors. The gut microbiota, encompassing both bacteria and fungi, contributes not only to digestion and metabolism but also to the synthesis of functional substances, playing a crucial role in immune regulation. Furthermore, its metabolites, such as short-chain fatty acids (SCFAs) and bile acids, affect intestinal neurosignaling, energy metabolism, immune functionality, and intestinal barrier function, thereby modulating the gut ecosystem in various ways (5–7). It is widely thought that the dynamic balance of these microorganisms is fundamental to intestinal homeostasis, and any disruption to this balance may lead to the onset and progression of disease.

Aside from the gut microbiota, the function of the intestinal tissue itself also plays a critical role in the occurrence and progression of diseases. The primary protective barrier in the intestinal lumen consists of intestinal epithelial cells, including mucus-secreting goblet cells and intestinal stem cells (ISCs), which are located at the base of the crypts. Collectively, these cells form a physical barrier through tight junctions (TJs), separating the intestinal lumen contents from the underlying tissue and preventing harmful substances from infiltrating the intestinal immune system, which encompasses both innate and adaptive immune cells, as well as the circulatory system (8). Besides, enzymes secreted by the gastrointestinal tract, along with glycoproteins and other chemical substances, constitute a chemical barrier that resists the invasion of microorganisms and harmful agents within the intestinal lumen, thereby preventing leakage and inhibiting pathogen growth (9).

Under physiological conditions, gut microorganisms and their metabolites maintain gut microenvironmental homeostasis through various mechanisms, including microbial balance, metabolic regulation, enhancement of barrier structure and function, and modulation of immune responses (10–12). These processes are accomplished via multiple pathways, including tight junction, leak, and unrestricted pathways (13). However, during disease progression, gut dysbiosis and the presence of harmful metabolites can compromise the intestinal barrier, leading to leakage into the submucosa and bloodstream (14). This damage activates immune cells, including dendritic cells (DCs) and macrophages, which subsequently initiate immune responses via various signaling pathways, such as Toll-like receptors (TLRs) acting as pattern recognition receptors (15). The subsequent release of inflammatory cytokines exacerbates tissue damage, further disrupts the intestinal barrier, and intensifies the imbalance (15). This cycle is perpetuated by feedback loops, leading to persistent disruption of local intestinal homeostasis. Furthermore, this dysregulation may trigger systemic diseases such as metabolic syndrome, non-alcoholic fatty liver disease, and diabetes by disrupting various signaling pathways in the liver, muscle, and adipose tissues (16, 17).

**Figure 1:**
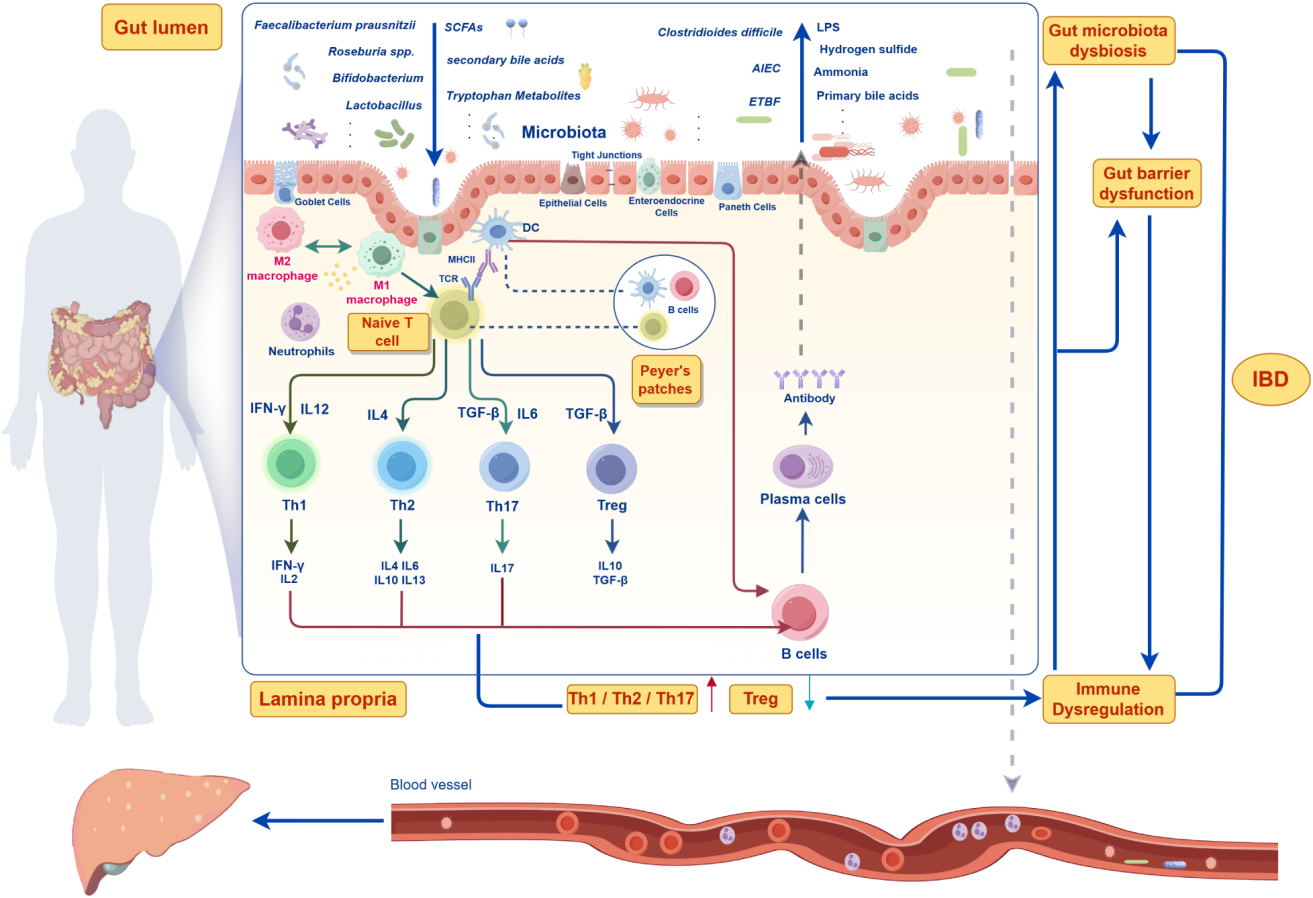
Pathophysiological mechanisms of gut microenvironment systemic dysregulation in inflammatory bowel disease (IBD).

Despite significant progress in recent years, research on the gut microenvironment has primarily focused on individual components or pathways, lacking a comprehensive analysis of the entire microenvironment. This approach poses challenges in comparing different studies and determining whether a specific change is causally related to disease outcomes. Moreover, existing therapeutic approaches, such as antibiotics, probiotics, and microbiota transplantation, exhibit variability in effectiveness across individuals and may carry potential risks (18). Therefore, this study investigates the gut microenvironment as a dynamic and complex entity, aiming to elucidate the interactions and regulatory mechanisms among microorganisms and their metabolites, epithelial cells, immune cells and signaling molecules in relation to IBD. By providing a holistic perspective, this review aims to offer new insights into IBD pathogenesis and establish a crucial theoretical foundation and practical guidance for the development of more effective therapeutic strategies in the future (**Figure 1**).

## 2 The dysregulation of gut microbiota and their metabolites

It is well-established that the pathogenesis of IBD is closely associated with the dysregulation of gut microbiota and their metabolites. In recent years, the Western diet, characterized by a high fat and protein content and low dietary fiber, along with the widespread use of food additives due to industrialization, has significantly altered both the structure and function of the gut microbiome (19, 20). This disruption, compounded by genetic susceptibility and environmental stressors, has emerged as a key trigger for the onset of IBD (21). It is now understood that under physiological conditions, beneficial gut bacteria, such as *Faecalibacterium prausnitzii, Roseburia* spp.*, Bifidobacterium*, and *Lactobacillus*, metabolize dietary components to produce SCFAs, including acetate, propionate, and butyrate, as well as indole metabolites and antimicrobial peptides. These metabolites play a crucial role in maintaining intestinal barrier integrity and immune homeostasis (22). For instance, SCFAs provide energy to intestinal epithelial cells, enhance the expression of TJ proteins (e.g., Zonula Occludens-1 (ZO-1) and Occludin), regulate immune responses by promoting regulatory T cell expansion, and inhibit inflammation (23–25). Besides, indole metabolites promote barrier repair and immune modulation by activating the aryl hydrocarbon receptor (AhR), while antimicrobial peptides further protect the intestinal barrier by inhibiting the growth of pathogenic bacteria (26, 27).

**Figure 2:**
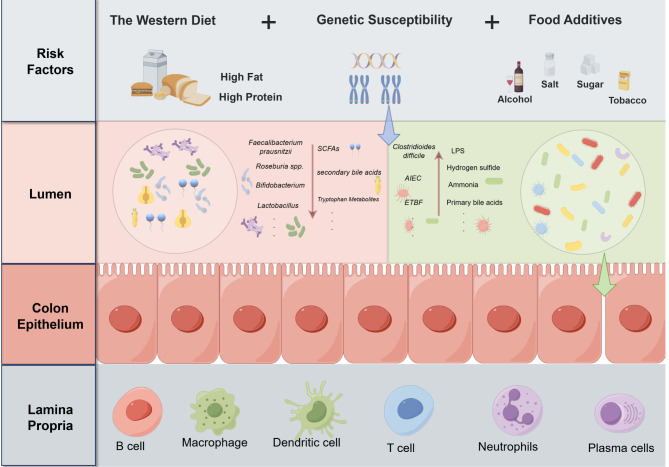
Risk factors driving the dysregulation of gut microbiota and their metabolites in IBD pathogenesis.

In contrast, patients with IBD exhibit significantly reduced gut microbiota diversity and a decreased abundance of beneficial bacteria, resulting in diminished capacity to produce SCFAs and indole metabolites (28). This reduction weakens the anti-inflammatory functions and barrier repair abilities of the gut. Concurrently, the abundance of pathogenic bacteria, such as Adherent-Invasive *Escherichia coli (AIEC), Enterotoxigenic Bacteroides fragilis (ETBF) and Clostridioides difficile*, is significantly increased (29). Toxins secreted by these bacteria (e.g., colibactin and *Bacteroides fragilis* toxin (BFT)) and their metabolic byproducts (e.g., lipopolysaccharides (LPS), hydrogen sulfide, and ammonia) disrupt the gut microenvironment in multiple pathways (30, 31). These harmful metabolites can directly damage epithelial cells, inducing apoptosis or necrosis, and can also activate pro-inflammatory signaling pathways (e.g., NF-κB) by binding to Toll-like receptor 4 (TLR4). This activation promotes the release of pro-inflammatory cytokines (e.g., IL-6, IL-17, and TNF-α), which directly disrupt TJ proteins, leading to increased intestinal permeability and exacerbating inflammation (32). Furthermore, harmful metabolites, including LPS, inhibit the growth of beneficial bacteria by altering the gut microenvironment (e.g., lowering pH and changing redox states) while simultaneously creating favorable conditions for the proliferation of pathogenic bacteria (33). Dysregulation of bile acid metabolism, characterized by an accumulation of primary bile acids and a decrease in secondary bile acids, further disrupts the balance of the gut microbiota and enhances pro-inflammatory effects (34). Thus, the imbalance of microorganisms and their metabolites constitutes a significant driver in the onset and progression of IBD (**Figure 2**).

In response to the aforementioned pathological conditions, various innovative therapeutic strategies have been proposed. Probiotics, as classical regulators of gut microbiota, have emerged as a significant approach in the treatment of IBD given their ability to restore the balance of gut microecology and exert anti-inflammatory effects. Studies have demonstrated that specific probiotics, such as *Lactobacillus* and *Bifidobacterium*, can significantly alleviate clinical symptoms of IBD by inhibiting the release of pro-inflammatory cytokines, enhancing gut barrier function, and providing antioxidant effects (35, 36). Clinical data indicate that probiotics demonstrate significant efficacy in achieving remission in ulcerative colitis (UC), with higher rates observed in the probiotic group compared to the placebo group (37). However, the overall effectiveness of probiotics remains a subject of debate. Some studies suggest that probiotics may not demonstrate significant differences in inducing remission when compared to placebo or standard treatments (38). Fecal microbiota transplantation (FMT) has emerged as an alternative therapeutic method for restoring gut microbiota diversity, utilizing microbiota derived from healthy donors to effectively modulate gut immune responses and reduce pathological inflammation. Studies have reported therapeutic efficacy rates ranging from 60% to 80%, with long-term remission rates around 50% (39). Despite the notable efficacy of FMT, challenges remain, including complexities associated with donor screening, potential infection risks during the treatment process, and variability in effectiveness due to individual patient differences. In summary, although these therapeutic strategies have made positive strides in improving IBD symptoms, they continue to face challenges related to variability in efficacy, side effects, and the demand for personalized treatment. Future research should focus on precisely modulating the gut microbiota to further optimize treatment regimens and provide patients with more accurate and personalized therapeutic strategies.

Collectively, these findings demonstrate that gut dysbiosis, characterized by a reduction in beneficial bacteria and an increase in pathogenic species, fundamentally disrupts the intestinal metabolic landscape, leading to a cascade of harmful compounds that compromise barrier integrity and immune homeostasis. While current therapeutic interventions targeting microbial restoration show promise, they require further optimization to address inter-individual variability and achieve sustained clinical efficacy.

## 3 Injury to the intestinal barrier

The intestinal barrier serves as a fundamental defense system, maintaining gut homeostasis and preventing the invasion of external pathogens and harmful substances. It is composed of both physical and chemical barriers. The physical barrier is mainly composed of intestinal epithelial cells, which establish a protective layer through TJ proteins such as Occludin, Claudins and ZO-1 that separate luminal contents from the underlying lamina propria (23). This separation helps prevent harmful substances from leaking into the intestinal immune system and bloodstream, both of which contain innate and adaptive immune cells. The self-renewal of intestinal epithelial cells relies on ISCs located in the intestinal crypts between the intestinal villi, which play a central role in maintaining the intestinal barrier. ISCs proliferate to generate progeny cells; some of these continue to function as ISCs to maintain their proliferative capacity, while others differentiate into functional epithelial cells, including absorptive enterocytes, goblet cells and enteroendocrine cells (40, 41). These differentiated cells are indispensable for normal intestinal physiology. In this respect, absorptive enterocytes are responsible for nutrient absorption and transport while maintaining the integrity of the intestinal barrier. Intestinal endocrine cells regulate multiple physiological functions of the gut by secreting hormones. Besides, goblet cells secrete mucus, which protects the intestinal epithelial barrier and prevents the translocation of harmful external substances (42).

Current evidence suggests that the processes of proliferation and differentiation of these stem cells are precisely regulated by multiple signaling pathways, particularly the Wnt, Notch, and Hedgehog pathways, which play crucial roles in the renewal of ISCs (43). Specifically, the Wnt signaling pathway not only maintains the undifferentiated state of ISCs and promotes their self-renewal, but also creates a conducive proliferative microenvironment within the intestinal glands (44). Moreover, Wnt signaling regulates downstream transcription factors that determine the differentiation of ISCs into various epithelial cell types (45). Besides, Notch signaling, through interactions between its receptor and ligand, activates intracellular transcription factors that maintain the proliferative capacity of ISCs (46). It also regulates the differentiation process via negative feedback mechanisms. Notably, during the formation of endothelial and goblet cells, Notch signaling interacts with the Wnt signaling to finely regulate the directional differentiation of ISCs (47). Hedgehog signaling activates Gli transcription factors to govern the proliferation and differentiation of ISCs, thereby playing a crucial role in maintaining their proliferative potential (48). Furthermore, Hedgehog signaling pathways coordinate with other pathways to regulate the three-dimensional structure and functionalization of tissues, promoting the directional differentiation of ISCs into specific cells and ensuring the intestinal barrier integrity (49). In addition to these molecular signals, mechanical signals significantly influence the differentiation of ISCs. The stiffness, tensile forces, and shear stress of the extracellular matrix (ECM) regulate cell morphology and migration through integrin-matrix interactions, ultimately affecting their differentiation. Studies have shown that mechanical characteristics, such as stiffness, tensile force, and shear stress, of the ECM can significantly influence cell morphological changes and migration through the interaction between integrins and the matrix, thereby regulating the differentiation of ISCs (50). Specifically, integrins, as the primary connecting molecules between cells and the extracellular matrix, can sense and respond to the mechanical properties of the matrix. The interaction between integrins and ECM not only affects cell adhesion and morphology but also regulates cell proliferation and differentiation by activating downstream signaling pathways. For instance, the binding of integrins to ECM can activate signaling pathways such as FAK and ERK, which play a crucial role in cellular mechanosignal transduction (51). In addition, the mechanical properties of the extracellular matrix regulate cell fate by influencing the reorganization of the cytoskeleton. The reorganization of the cytoskeleton can alter the shape and mechanical characteristics of cells, thereby affecting their differentiation direction. Current evidence suggests that the interplay of mechanical and physiological signals within the microenvironment collectively determines the differentiation fate of ISCs. This regenerative process not only ensures the continuous turnover of the intestinal epithelium but also facilitates barrier repair following injury. Following damage to the intestinal epithelium, ISCs proliferate and differentiate into new functional cells. This process facilitates the repair of the intestinal barrier and restores its physical and chemical defense functions. Notably, upon differentiating into goblet cells, ISCs secrete mucus, forming a physical barrier in the gut that prevents the invasion of harmful substances and pathogens. Furthermore, ISCs contribute not only to local barrier repair but also to the adaptive regulation within the gut microenvironment, aiding in the maintenance of gut homeostasis and immune balance. Dysregulation of ISC function or abnormal proliferation and differentiation can compromise barrier integrity, contributing to gut pathology and triggering various intestinal diseases, such as leaky gut syndrome and IBD (52). The rapid renewal of intestinal epithelial cells enhances the dynamic repair capacity, effectively maintaining the structural integrity of the barrier.

The chemical barrier is a crucial defense system in the gut, with one of its key components being the mucus layer that coats the epithelial surface. This layer serves as the first line of defense, protecting gut homeostasis by blocking pathogens from contacting the epithelial surface while providing a habitat for the commensal microbiota (53). Studies have found that the intestinal mucus layer in patients with IBD is significantly thinner or locally absent, which not only exposes epithelial cells directly to the contents of the intestinal lumen but also substantially increases the risk of pathogen colonization (54). Furthermore, the secretion of antimicrobial peptides (e.g., defensins) by Paneth cells and epithelial cells is often diminished in IBD patients, further weakening the protective function of the chemical barrier (55). In addition to the physical and chemical barriers, the immune system also plays a defensive role in maintaining gut homeostasis. Innate immune cells (e.g., macrophages and DCs) and adaptive immune cells (e.g., T cells) initially secrete cytokines to exert anti-inflammatory effects during the gut’s initial inflammatory state. However, the ongoing occurrence and progression of inflammation can drive the persistent release of pro-inflammatory factors (e.g., TNF-α, IL-6, and IL-17), which exacerbate intestinal inflammation and inhibit the barrier’s repair functions (56, 57).

**Figure 3:**
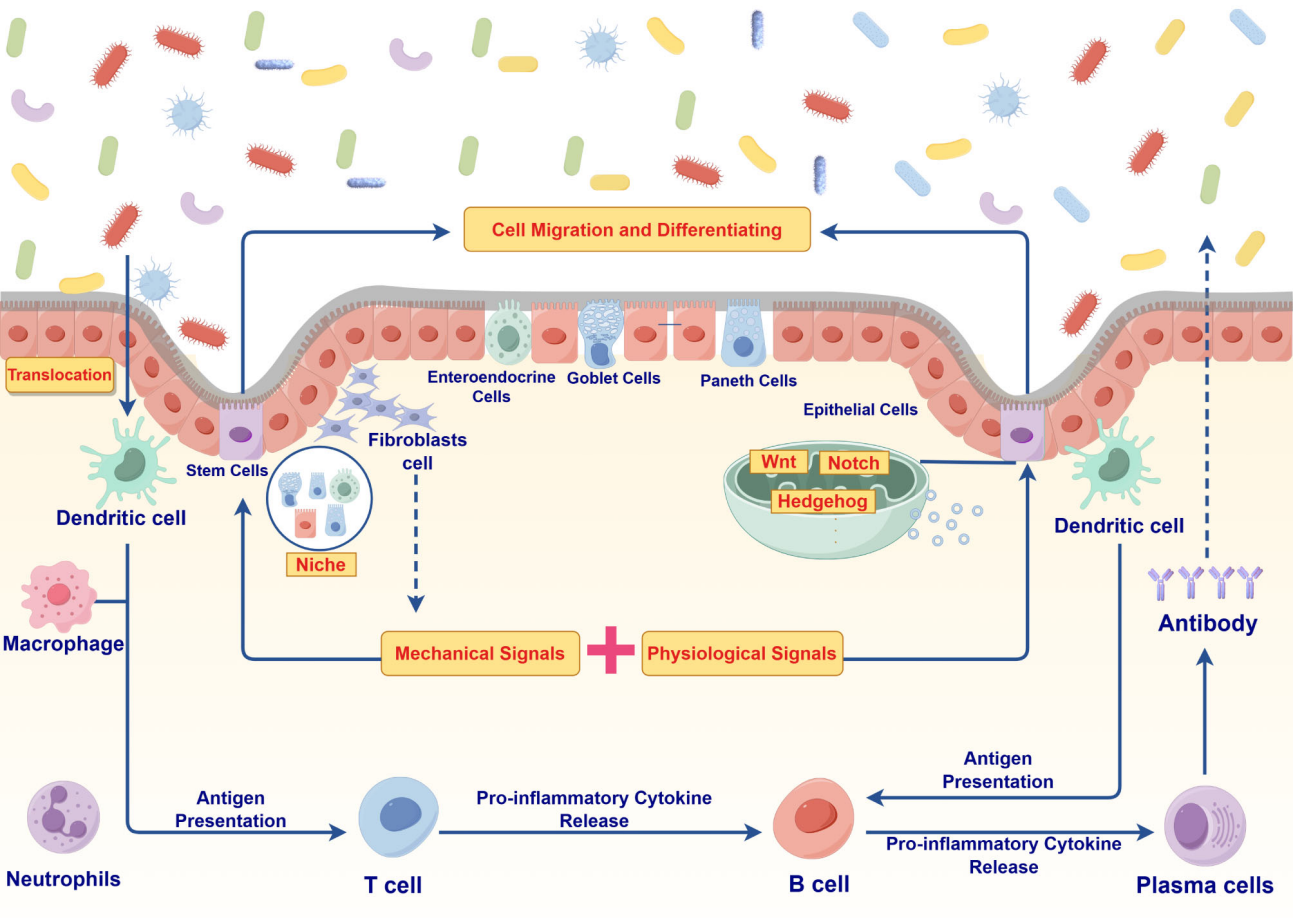
Mechanism of intestinal barrier dysfunction and ISCs regulation in IBD pathogenesis.

In patients with IBD, gut dysbiosis and its metabolites can directly damage the physical and chemical barriers of the gut, leading to epithelial injury and a reduction in TJ protein expression, which is considered one of the primary causes of increased intestinal permeability (58). Damage to the intestinal barrier allows toxic metabolites and microorganisms to easily cross the barrier and leak into the lamina propria or the bloodstream. This leakage activates immune cells such as macrophages, DCs, and T lymphocytes, disrupting the immune barrier and inducing the release of additional pro-inflammatory factors, which further impair barrier function. Moreover, the leaked toxins (e.g., LPS) may cause metabolic disorders (including insulin resistance and abnormalities in lipid metabolism) through systemic circulation, exacerbating the systemic inflammatory state of IBD and worsening damage to the intestinal barrier (59, 60). Consequently, this impairment of barrier function leads to significant leakage of intestinal contents, providing toxic metabolites that contribute to the characteristic persistent inflammation associated with IBD (**Figure 3**).

Current therapeutic strategies for intestinal barrier damage primarily focus on improving gut barrier function, reducing intestinal permeability, and promoting the repair of intestinal epithelial cells. For instance, probiotic therapy can restore the balance of gut microbiota and facilitate the repair of the intestinal barrier. SCFAs, as metabolic products of gut microbiota, play a crucial role in barrier repair by promoting TJ formation in intestinal epithelial cells and exerting anti-inflammatory effects. Besides, pharmacological agents such as glutamine and other gut protectants directly act on intestinal epithelial cells to enhance gut barrier function and improve clinical symptoms in patients with IBD (61). Despite the significant effectiveness of these treatment strategies in clinical practice, challenges remain, including interindividual variability in efficacy, potential side effects, and issues with drug tolerance, highlighting the need for further research to optimize treatment approaches.

Overall, intestinal barrier dysfunction in IBD encompasses multiple interconnected components, comprising compromised stem cell regeneration, disrupted tight junction integrity, reduced chemical barrier protection, and impaired immune surveillance. Therefore, effective therapeutic strategies require integrated approaches that simultaneously target these multiple barrier elements rather than focusing on isolated pathways.

## 4 Activation of the immune response

A compromised intestinal barrier can allow microorganisms and their harmful metabolites to leak into the lamina propria. This leakage triggers complex immune responses that may contribute to inflammatory processes and various other health issues.

### 4.1 Innate immune response

Once the integrity of the intestinal barrier is compromised, microorganisms and their metabolites translocate across the epithelium, prompting a rapid and coordinated innate immune response. This process involves the activation and interplay of various immune cells, orchestrating a dynamic and precise immune defense mechanism. Among the first responders are the resident macrophages within the lamina propria, which quickly recognize pathogen-associated molecular patterns (PAMPs) and damage-associated molecular patterns (DAMPs) through pattern recognition receptors (PRRs) on their surface, including TLRs and NOD-like receptors (NLRs). TLRs can recognize microbial components such as LPS and double-stranded RNA, while NLRs recognize bacterial peptidoglycans (62, 63). Upon activation, macrophages secrete various pro-inflammatory cytokines (e.g., IL-6, TNF-α, and IL-1β) to initiate local inflammation and release the chemokine CXCL8 to attract more immune cells to the site of infection (64). Moreover, macrophages phagocytose and eliminate pathogens while releasing reactive oxygen species (ROS) and nitrogen oxides (NO) to further facilitate pathogen clearance (65). By performing these functions, macrophages process antigen fragments and present them on MHC II molecules, thereby bridging innate and adaptive immunity. Moreover, under the influence of different microenvironmental signals, macrophages can polarize into M1 or M2 subtypes. M1 macrophages secrete IL-12 and TNF-α to enhance inflammatory responses and activate Th1 and Th17 immunity, while M2 macrophages secrete IL-10 and TGF-β to promote tissue repair and immune regulation and activate Treg immunity (66). Indeed, this equilibrium between M1/M2 polarization reflects the dual roles of macrophages in immune responses. In a healthy state, a dynamic balance between M1 and M2 macrophages is maintained, ensuring a rapid response to pathogen invasion while preventing excessive inflammatory responses (67).

However, during the onset and progression of IBD, this balance is disrupted. Persistent exposure to microorganisms and metabolites in the intestinal microenvironment drives the overactivation of M1 macrophages. Their continuous secretion of high levels of pro-inflammatory factors not only sustains and exacerbates local inflammatory responses but may also cause damage and apoptosis of intestinal epithelial cells, further compromising gut barrier function and creating a deleterious feedback loop (68). At the same time, the anti-inflammatory and repair functions of M2 macrophages are limiting their ability to counteract M1 macrophage overactivation and the excessive secretion of inflammatory factors. Emerging evidence suggests that macrophages in IBD patients may exhibit impaired polarization toward the M2 phenotype or may exhibit functional abnormalities during the polarization process, resulting in insufficient anti-inflammatory and repair responses (69). Moreover, the chronic inflammatory milieu promotes oxidative stress and the continuous release of cytokines, enhancing the M1 macrophage phenotype while recruiting additional immune cells, such as T cells and neutrophils, into the inflammatory cascade, creating a self-sustaining chronic inflammatory microenvironment that promotes the persistence and exacerbation of IBD.

Neutrophils are the key effectors of the secondary immune wave that follows macrophage activation. Attracted by chemokines (e.g., CXCL8) and pro-inflammatory factors (e.g., TNF-α and IL-1β) secreted by macrophages and epithelial cells, neutrophils are swiftly recruited to the sites of infection within hours following intestinal barrier disruption (70). These innate immune cells exhibit antimicrobial function and utilize ROS and antimicrobial enzymes (e.g., myeloperoxidase and elastase) to clear pathogens. Besides, neutrophils can capture and contain pathogens through the formation of neutrophil extracellular traps (NETs), which comprise decondensed chromatin and antimicrobial proteins (71). This process further enhances local defense mechanisms by immobilizing pathogens and preventing their spread. While these processes are crucial for defense, the release of these cytotoxic molecules can cause damage to surrounding tissues, exaggerating inflammation and contributing to the chronic IBD (72).

Building on the roles of macrophages and neutrophils, DCs have gradually emerged as a key bridge connecting innate and adaptive immunity. In response to microenvironmental signals (e.g., IL-1β and TNF-α), DCs migrate to the sites of injury, where they recognize and capture pathogens and their metabolites through PRRs. Similar to macrophages, DCs can process antigens through phagocytosis; however, their distinct capability lies in their ability to present processed antigens to naïve T cells via MHC II molecules (73). During this process, DCs undergo several steps, including antigen uptake, intracellular processing, upregulation of surface molecules, migration to lymphoid tissues, effective interaction with T cells and regulation of T cell differentiation. Upon encountering pathogens or inflammatory signals (e.g., bacterial components, IL-1β, and TNF-α), DCs become activated and undergo maturation (74). During this transition, MHC II molecules are significantly upregulated to enhance antigen presentation capabilities, and the expression of co-stimulatory molecules CD80 (B7-1) and CD86 (B7-2) increases. This interaction with CD28 on T cells provides the necessary second signal for T cell activation, thereby preventing ineffective or spontaneous immune responses (75). Furthermore, the upregulation of the chemokine receptor CCR7 directs mature DCs to migrate to secondary lymphoid organs (e.g., Peyer’s patches), facilitating contact with T cells (76). During antigen processing, DCs capture pathogens or their metabolites through phagocytosis, pinocytosis, or receptor-mediated endocytosis. They degrade these materials into short peptide fragments and transport them to the cell surface after binding to MHC II molecules for recognition by T cells. In secondary lymphoid tissues, DCs present antigen peptides via MHC II molecules while providing co-stimulatory signals by binding CD80/CD86 to CD28 on T cells, thereby promoting the comprehensive activation and proliferation of T cells (77). Current evidence suggests that DCs secrete cytokines such as IL-12 and IL-23, which guide T cell differentiation into various subtypes, including pro-inflammatory Th1, Th17, and anti-inflammatory regulatory T cells (Treg) (78). DCs not only activate T cells but can also induce T cell apoptosis or drive Treg generation to prevent autoimmune responses (81). This function is particularly crucial for maintaining homeostasis in immune organs such as the gut. However, in diseases such as IBD, DCs may experience functional disturbances, including overactivation leading to excessive secretion of pro-inflammatory cytokines, abnormal antigen presentation that activates self-reactive T cells, and loss of regulatory functions that result in the erosion of immune tolerance (82, 83).

**Figure 4:**
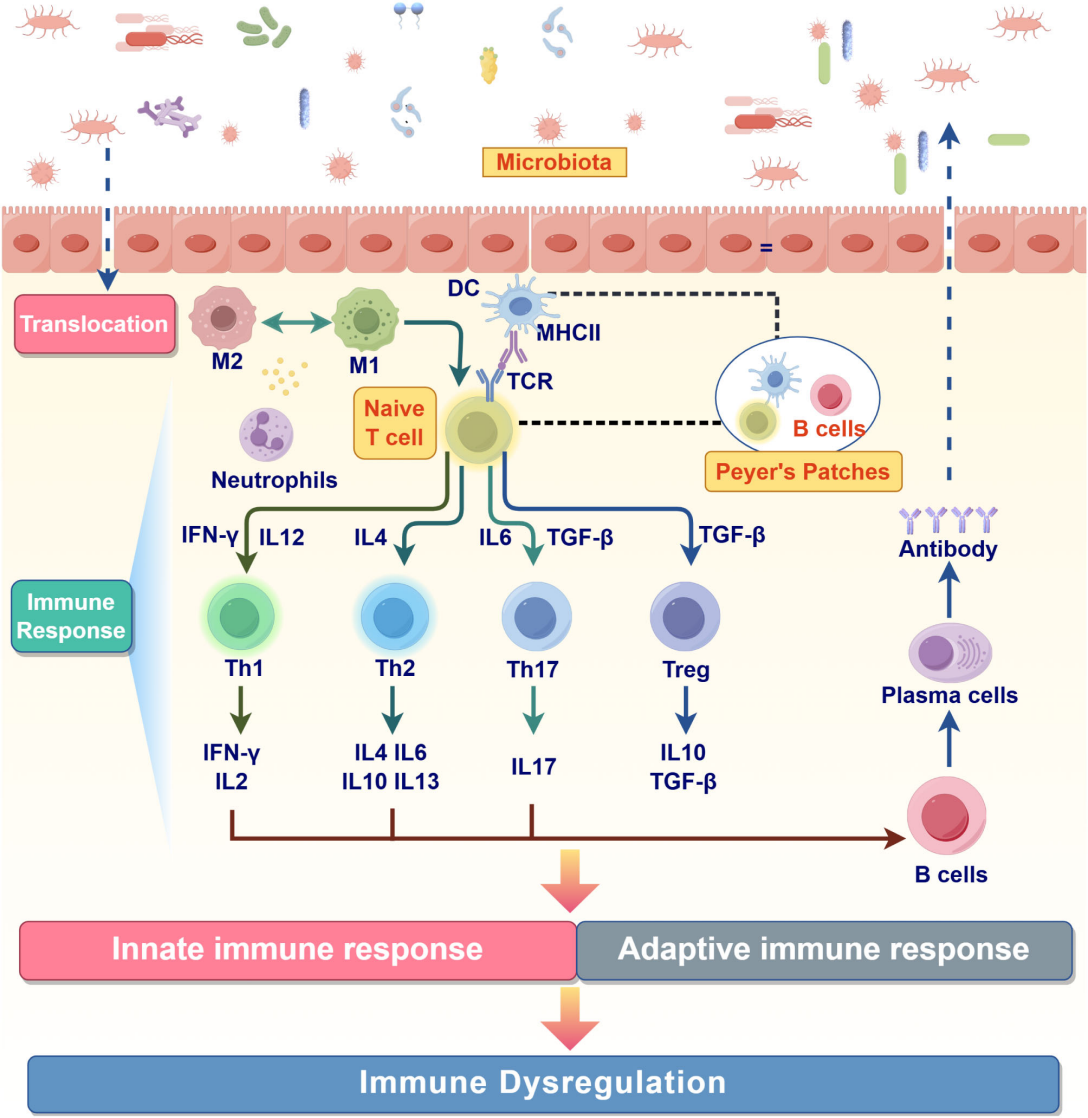
Dysregulation of innate and adaptive immune responses in IBD pathogenesis.

Macrophages serve as the first line of defense in the innate immune response, rapidly containing the spread of pathogens through pattern recognition, the secretion of inflammatory factors, and phagocytosis, thereby establishing a foundation for adaptive immunity. Neutrophils are rapidly recruited to the site of infection, where their primary function is the swift elimination of pathogens, thereby playing a critical role in the early stages of infection. The involvement of DCs marks the transition from innate to adaptive immunity, as they integrate the entire immune defense system through antigen presentation and the secretion of cytokines. The successive activation and functional cooperation of these three cell types create a precisely timed and functionally complementary immune network capable of swiftly controlling pathogens while ensuring the subsequent specific immune response. However, in the pathological process of IBD, pro-inflammatory Th1 and Th17 responses exhibit sustained activation, while anti-inflammatory Treg responses are often insufficient to fully suppress inflammation, leading to a sustained inflammatory response that cannot be effectively controlled. This chronic inflammation is further exacerbated by the dysregulation of macrophage polarization. The failure to restore homeostasis results in a self-perpetuating inflammatory cycle that drives disease onset and progression (**Figure 4**).

In conclusion, macrophages in intestinal immunity exhibit both pro-inflammatory and anti-inflammatory repair functions. The onset and progression of IBD arise from an imbalance between these functions, where the pro-inflammatory role predominates while the anti-inflammatory and repair functions are insufficient, leading to persistent inflammation and tissue damage in the gut. Moreover, DCs further exacerbate this imbalance by influencing the development of T cell subtypes. Therefore, therapeutic strategies aimed at regulating the functions of macrophages and DCs, such as promoting the polarization of M2 macrophages and Treg, or inhibiting the overactivation of M1 macrophages and Th1/Th17 cells, have emerged as promising directions in current IBD research and treatment.

In summary, the dysregulated innate immune response in IBD is characterized by persistent M1 macrophage activation, excessive neutrophil recruitment, and aberrant dendritic cell function, creating a self-perpetuating inflammatory cycle. Indeed, disrupting this pathological loop requires therapeutic strategies that can simultaneously restore immune cell balance and address the underlying microenvironmental triggers.

### 4.2 Adaptive immune response

The initiation of the adaptive immune response begins with the presentation of antigens to naïve T cells, leading to their successful activation and differentiation. This crucial process is primarily orchestrated by professional antigen-presenting cells (APCs), especially DCs (84). Through the sequential processes of antigen uptake, intracellular processing, surface molecule upregulation, migration to lymphoid tissues, and effective interaction with T cells, DCs establish the fundamental antigen-MHC presentation and co-stimulatory signaling required for T cell activation and subsequent differentiation regulation (85, 86).

Upon activation, naïve T cells undergo clonal expansion and differentiate into various effector T cell subtypes (Th1, Th2, Th17, Treg) or memory T cells, executing specific immune functions. The cytokines released by DCs play a crucial role in the differentiation pathways of T cells. Importantly, DCs can regulate the directional response of the immune system through the dynamic equilibrium of their subtypes. In this regard, in the gut of healthy individuals, tolerogenic DCs in the gut secrete IL-10 and TGF-β to induce the differentiation of naïve T cells into Treg, sustaining mucosal immune homeostasis (87). Conversely, pro-inflammatory DCs secrete IL-12 and IL-23 to drive the differentiation of Th1 and Th17 cells exacerbating inflammation (88). For instance, under the influence of IL-12, Th1 cells secrete IFN-γ to counter intracellular pathogens; under IL-23, Th17 cells secrete IL-17 to participate in inflammatory responses against fungi and bacteria (79). Meanwhile, Treg cells differentiate under the regulation of TGF-β and IL-10, secreting anti-inflammatory factors to suppress excessive inflammation and maintain immune tolerance (80). Furthermore, when DCs secrete IL-6 in synergy with IL-1β or IL-23, they promote Th17 cell differentiation (89). In the absence of IL-23, however, they support the proliferation and differentiation of B cells, ultimately facilitating the generation of plasma cells that produce antibodies to neutralize pathogens or activate the complement system (90). Under specific conditions, DCs can also secrete low levels of IL-4, which supports the differentiation of Th2 cells during parasitic infections or allergic responses. The differentiated Th2 cells secrete IL-4, IL-5, and IL-13, inducing IgE production in B cells while activating eosinophils to eliminate parasites and enhancing mucus secretion to protect the epithelial barrier (91).

DCs initiate the immune response by activating naïve T cells via antigen presentation and cytokine secretion. These newly differentiated effector T cells then secrete specific cytokines to recruit and activate macrophages, neutrophils, B cells, and natural killer (NK) cells, forming a collaborative immune network. This complex and dynamic interplay within the adaptive immune system ensures the effective elimination of pathogens while rigorously maintaining self-tolerance to prevent autoimmunity.

However, in patients with IBD, this balance is disrupted, resulting in a significant increase in pro-inflammatory DCs that excessively secrete IL-12 and IL-23. This leads to the abnormal expansion and activation of Th1 and Th17 cells, creating a persistent pro-inflammatory microenvironment (92). As key effector cells in IBD pathogenesis, activated Th1 and Th17 cells are the primary effector cells in the pro-inflammatory response in IBD. Th1 cells are now understood to enhance the phagocytic and bactericidal abilities of M1 macrophages through the secretion of IFN-γ and TNF-α while also promoting cytotoxic responses mediated by CD8+ T cells (93). However, the overactivity of Th1 cells can contribute to intestinal tissue damage (94). It has been reported that Th17 cells primarily secrete IL-17 and IL-22, with IL-17 recruiting neutrophils to eliminate extracellular pathogens, which can lead to excessive inflammation and tissue destruction. Under physiological conditions, IL-22 promotes the repair of the epithelial barrier; however, in the context of IBD, dysregulated IL-22 signaling can exacerbate intestinal barrier dysfunction (95–97). Furthermore, a synergistic interaction between Th1 and Th17 cells amplifies the pro-inflammatory cascade, contributing to the chronic and often irreversible nature of intestinal inflammation.

Conversely, anti-inflammatory Tregs play a crucial role in suppressing the activity of Th1 and Th17 cells, balancing pro-inflammatory responses and maintaining immune homeostasis through the secretion of IL-10 and TGF-β. However, in patients with IBD, the reduction in Treg cell abundance or their functional impairment significantly contributes to uncontrolled inflammation (98). Notably, the differentiation of Treg and Th17 cells is regulated by common signals such as TGF-β. Under physiological conditions, TGF-β and IL-6 work synergistically on naïve T cells to induce Th17 differentiation (99). However, in environments lacking IL-6 or rich in IL-10, TGF-β tends to favor Treg differentiation (100). A study on IBD patients revealed that excessive secretion of IL-6 and insufficient expression of IL-10 could significantly enhance Th17 differentiation while suppressing Treg differentiation (101, 102). The disruption in the Th1 and Th17 axis represents a core pathological mechanism underlying the development of IBD.

Furthermore, in IBD, the role of plasma cells in antibody secretion exhibits a complex duality, where this dysfunctional function may exacerbate inflammatory responses and interfere with the integrity and repair capacity of the intestinal barrier. Under normal circumstances, IgA antibodies maintain gut homeostasis by preventing pathogen adhesion and clearing harmful microorganisms. However, in patients with IBD, the types of antibodies secreted by plasma cells may become abnormal, marked by excessive secretion of IgG antibodies. Excessive IgG binds to pathogens or intestinal microbiota, forming immune complexes that activate the classical complement pathway and lead to C3a and C5a release, which recruit neutrophils and monocytes, thereby intensifying the inflammatory response (103, 104). Moreover, IgG can bind to Fcγ receptors on the surface of macrophages or DCs, inducing the secretion of numerous pro-inflammatory factors, such as TNF-α and IL-6, which further amplify inflammation. In some cases, IgG may target intestinal self-antigens, leading to autoimmune damage. This abnormal pro-inflammatory environment is detrimental to inflammation control and, through complement-mediated membrane attack complexes (MAC), directly damages epithelial cells. This damage exacerbates barrier permeability, allowing intestinal microbes and endotoxins to translocate across the barrier. This influx further activates the immune system, creating a deleterious feedback loop of inflammation (105–107).

Interestingly, antibodies secreted by plasma cells also affect the repair of the intestinal barrier through various mechanisms. Complement activation and Fcγ receptor signaling can induce apoptosis or necrosis of epithelial cells, leading to the loss of TJ proteins and thereby weakening barrier function (108, 109). In addition, IL-13 secreted by Th2 cells in patients with IBD not only promotes plasma cell-driven secretion of IgE antibodies but also suppresses the regenerative capacity of epithelial cells, further exacerbating the challenges to intestinal barrier repair in conjunction with the damage caused by complement activation (110, 111). Moreover, this pro-inflammatory environment and abnormal antibody secretion significantly impair the regenerative capacity of intestinal epithelial stem cells, which are essential for maintaining the integrity and self-repair of the intestinal barrier. Excessive production of pro-inflammatory factors (e.g., TNF-α, IL-6, and complement C3a) inhibits the proliferation and differentiation of ISCs by activating the NF-κB and STAT3 signaling pathways. Meanwhile, complement-mediated inflammatory mediators (e.g., C5a) recruit neutrophils and monocytes, which release ROS and proteases, causing direct damage to ISCs and their niche (112). Furthermore, IL-13, through specific signaling pathways, not only inhibits the proliferation of stem cells but may also alter their differentiation direction, making them more prone to differentiate into goblet cells rather than absorptive epithelial cells, thus impairing barrier repair functions (113).

Macrophages play a critical role in both innate and adaptive immunity. In response to persistent inflammation, the balance between M1 and M2 macrophage differentiation becomes increasingly disrupted. Moreover, the factors secreted by macrophages progressively participate in adaptive immunity. It has been established that M1 macrophages secrete pro-inflammatory cytokines, such as IL-1β, IL-6, and TNF-α, which engage in inflammatory crosstalk with Th1 and Th17 cells, thereby creating a self-reinforcing pro-inflammatory network. In patients with IBD, the proportion of M1 macrophages is significantly elevated, and their continuous activation leads to the overproduction of pro-inflammatory cytokines, thereby exacerbating tissue damage. Conversely, the insufficient number and impaired function of M2 macrophages restrict the anti-inflammatory response, undermining effective inflammation regulation.

The function of T cells and the polarization state of macrophages are significantly influenced by the intestinal microenvironment. For instance, SCFAs derived from a healthy microbiota, such as butyrate, induce M2 polarization and enhance the function of Treg cells. Conversely, in patients with IBD, dysbiosis leads to a decrease in SCFAs levels, impairing the immune regulatory functions of M2 macrophages and Tregs.

The regulation of adaptive immunity by the intestinal microenvironment is not limited to the action of cytokines but also involves the participation of gut microbiota and their metabolic products. A healthy microbiota can interact directly with DCs through metabolites such as SCFAs, promoting intestinal immune homeostasis. However, in IBD, the diversity of the gut microbiota is significantly decreased, and the overgrowth of specific pathogenic bacteria, such as mucosa-adhering *Escherichia coli*, directly stimulates DCs and M1 macrophages, resulting in the overproduction of pro-inflammatory cytokines. Furthermore, the deficiency of SCFAs in the microenvironment restricts the expansion and anti-inflammatory functions of Tregs, exacerbating the uncontrolled inflammatory response.

In summary, the dual roles of pro-inflammatory and anti-inflammatory responses in adaptive immunity are crucial for maintaining immune homeostasis. Excessive pro-inflammatory signaling coupled with inadequate anti-inflammatory counter-regulation can lead to the onset and progression of IBD. This mechanism involves not only the imbalance among Th1, Th17 and Treg cells and their associated cytokines but also includes the abnormal secretion of plasma cell antibodies, impaired proliferation and regenerative capacity of stem cells, dysfunction of macrophages in M1/M2 polarization, aberrant regulation of DCs during antigen presentation, and dysregulation of gut microbiota and their metabolites (**Figure 4**).

Therefore, targeting the balance between pro-inflammatory and anti-inflammatory mechanisms in adaptive immunity is a key objective in treating IBD. Current research indicates that selectively regulating pro-inflammatory factors and enhancing anti-inflammatory mechanisms demonstrates substantial therapeutic potential. For instance, anti-IL-12/IL-23 monoclonal antibodies, such as Ustekinumab, effectively reduce the proliferation of Th1 and Th17 cells. Besides, anti-TNF-α medications, such as Infliximab, alleviate inflammation by neutralizing key pro-inflammatory factors. Besides, supplementing microbial metabolic products, such as butyrate, or promoting the expression of IL-10 and TGF-β can enhance Treg function and M2 macrophage polarization, thereby restoring intestinal immune homeostasis (114). Emerging therapeutic strategies also include engineered dendritic cell therapy, which involves the *ex vivo* expansion of tolerogenic DCs and their reinfusion into patients to facilitate Treg expansion while suppressing Th1/Th17 activity, ultimately alleviating the inflammatory response.

Therapeutic strategies targeting the abnormal secretion of plasma cell antibodies in IBD also present considerable potential. On the one hand, B-cell depletion therapies, such as anti-CD20 monoclonal antibodies, can reduce the abundance of precursor B cells and diminish the secretion of abnormal antibodies. On the other hand, promoting IgA secretion while limiting IgG production, such as through the supplementation of short-chain fatty acids like butyrate, may effectively alleviate inflammation and restore barrier function. Besides, blocking Fcγ receptors or the complement system (e.g., C5 inhibitors) can reduce IgG-mediated pro-inflammatory responses while enhancing stem cell function and barrier repair by activating the Wnt signaling pathway or suppressing pro-inflammatory factors, such as TNF-α and IL-6. A comprehensive therapeutic strategy aims to regulate antibody secretion, protect the ISCs niche, and restore barrier integrity, thereby providing more precise and effective treatment options for patients with IBD.

In the future, enhancing the function and abundance of Treg cells can effectively suppress pro-inflammatory responses. Conversely, using specific inhibitors or neutralizing antibodies to reduce the production of Th1 and Th17 cells, along with their associated pro-inflammatory factors, will also be crucial. Restoring the balance of the immune system may involve targeting pro-inflammatory cytokines such as IL-12, IL-23, and IL-17, or enhancing the expression of anti-inflammatory factors like IL-10 and TGF-β. Moreover, promoting the polarization of M2 macrophages while inhibiting the excessive activation of M1 macrophages can help reduce inflammation and facilitate tissue repair. Through these comprehensive immune modulation strategies, the aim is to restore the balance of the immune system, alleviate intestinal inflammation, and promote tissue repair, thereby effectively controlling and relieving IBD. Conducting in-depth research into the interactions among Th1, Th17, Treg cells, B cells, plasma cells, antibodies, DCs, macrophages and the microenvironment, as well as developing precise modulation strategies based on these mechanisms, could lead to breakthroughs in IBD treatment.

Taken together, adaptive immune dysfunction in IBD involves a complex network of dysregulated responses: excessive Th1/Th17 activation, insufficient Treg suppression, abnormal antibody production, and impaired tissue regeneration. Effective intervention must address this multifaceted immune imbalance through precision targeting of interconnected cellular and molecular pathways.

### 4.3 Consequences of the immune response

In the immune system’s response to pathogens, the body may undergo different outcomes that can be categorized in order of severity, ranging from mild to severe. These outcomes are influenced by several factors, including the balance of the immune system, the nature of the pathogen and environmental conditions.

#### 4.3.1 Effective pathogen clearance and homeostasis restoration

When the immune system responds efficiently under the coordinated actions of innate and adaptive immunity, pathogens can be swiftly cleared, accompanied by effective tissue repair. Macrophages and neutrophils play a crucial role in reducing the pathogen load through phagocytosis and facilitating pathogen clearance. Meanwhile, DCs activate adaptive immunity by presenting antigens, which prompts effector T cells, such as Th1, Th2, and Th17, to mount functional immune responses. Following pathogen eradication, Tregs secrete anti-inflammatory cytokines such as IL-10 and TGF-β, which inhibit inflammation and promote homeostasis. Importantly, M2 macrophages further facilitate tissue repair, while the epithelial barrier is restored under the influence of IL-22 (115). Throughout this process, immune memory is established, enabling a rapid response to subsequent encounters with similar pathogens. Ultimately, the immune network returns to a resting state and homeostasis is restored within the microenvironment.

#### 4.3.2 Perpetuating inflammation reinforcing the chronic inflammatory conditions

If the immune response fails to resolve following pathogen clearance, or in the presence of persistent antigenic stimulation, chronic inflammation may ensue. A functional deficiency or insufficient abundance of Treg cells can cause the adaptive immune response to erroneously shift towards targeting self-antigens, resulting in autoimmune-mediated inflammation. Effector T cells may target intestinal epithelial cells or commensal microbiota, resulting in tissue damage (4). Aberrant immune activation is a hallmark of inflammatory bowel diseases. Crohn’s disease and ulcerative colitis are characterized by diffuse immune attacks on the intestinal mucosa, which exacerbate mucosal injury and compromise barrier integrity. Under these conditions, Th1 and Th17 responses are excessively active, with pro-inflammatory factors such as IFN-γ, IL-17, and TNF-α maintained at high levels for prolonged periods. This persistent activation drives continuous recruitment and activation of macrophages and neutrophils, which release ROS and proteases, resulting in repeated damage to intestinal tissues. Furthermore, the sustained recruitment of neutrophils and macrophages can further compromise the intestinal barrier’s integrity, exacerbating the inflammatory cycle. This persistent immune activation also disrupts the gut microbiota. Elevated levels of pro-inflammatory cytokines alter the gut’s pH and redox balance, suppressing beneficial microbes while promoting pathogenic bacterial overgrowth. This shift can lead to a long-term state of microenvironment dysbiosis, characterized by a decreased diversity of beneficial microbial species and an overgrowth of pathogenic bacteria. As the beneficial bacteria diminish, the production of SCFAs and other metabolites that facilitate gut health is reduced, further impairing the intestinal barrier and perpetuating inflammation. This dysbiotic state can contribute to a feedback loop of immune activation and further dysregulation, reinforcing the chronic inflammatory conditions. Ultimately, the interplay between immune dysfunction and gut microbiota dysbiosis drives the onset of chronic inflammatory disease, specifically IBD.

To summarize, immune response outcomes in IBD range from effective pathogen clearance to destructive chronic inflammation, with the gut microenvironment playing a critical role in determining these divergent pathways. Understanding this mechanistic framework provides essential insights for developing interventions that redirect immune responses toward homeostatic restoration.

## 5 Therapeutic approaches for IBD

According to the Global Burden of Disease (GBD) 2019 data, the standardized incidence rate of IBD in China increased from 1.47 per 100,000 in 1990 to 3.01 per 100,000 in 2019, marking a 104.76% increase (116). IBD represents a quintessentially progressive disorder characterized by gradual disease worsening and increasing complexity, where inadequately controlled intestinal inflammation leads to heightened risks of disease progression and complications (199). Epidemiological studies further substantiate the clinical significance of this progression, with progression to moderate-to-severe disease within one year of diagnosis in 24.5% of patients with mild UC and 46% of those with mild CD (200). This disease progression not only severely impairs patient quality of life but also significantly affects long-term clinical outcomes, with extraintestinal manifestations emerging as important determinants of morbidity and mortality in IBD patients (201). Given the profound impact of IBD progression on patient prognosis, establishing effective therapeutic strategies to control disease advancement and improve patient outcomes becomes critically important and urgent.

Current treatments of IBD primarily rely on pharmacotherapy supported by surgical intervention and adjunctive therapies. Despite achieving some efficacy in symptom improvement and induction of remission, these approaches remain insufficient for long-term disease control. Firstly, existing pharmacological treatments, including aminosalicylates, corticosteroids, immunomodulators and biologics, may alleviate symptoms but do not provide a complete cure. Actual remission rates range from only 30% to 60%, indicating that many patients do not achieve long-term disease control (117). Besides, many individuals undergoing anti-TNF therapy may experience reduced efficacy or complete ineffectiveness, underscoring the need for individualized and precise treatment strategies as a crucial area of research (118). Moreover, biologic therapy may develop extraintestinal manifestations such as oral ulcers, which further demonstrate the limitations of current treatment methods in achieving comprehensive disease management (119).

While surgery can alleviate symptoms to some extent, it is not curative and has several inherent limitations. First, preoperative nutritional optimization is a crucial factor in improving surgical outcomes. However, there is currently a lack of standardized protocols for preoperative optimization management (120). Second, many patients may still experience disease recurrence after surgery. Research indicates that patients with Crohn’s disease have a higher risk of postoperative recurrence, especially when effective medical management is not implemented following surgery (121). Besides, the surgery itself carries certain risks of complications, such as postoperative infections and bowel leakage, which can adversely affect recovery and overall quality of life (122). The timing of surgery and the selection of appropriate indications also present significant challenges. While early surgical intervention may lead to better outcomes in some cases, it is not indicated for all patients. Clinical decisions regarding surgical intervention should be individualized based on a comprehensive consideration of the severity of the patient’s condition, previous treatment responses, and potential risks and benefits of the procedure (123). Therefore, the application of surgical intervention should be conducted in collaboration with a multidisciplinary team to ensure that the best treatment plan is tailored to the personalized needs of each patient (124).

Adjunctive therapies, including probiotics, FMT and dietary modifications, are widely thought to yield beneficial effects in alleviating symptoms. These approaches aim to modulate the gut microenvironment, which plays a crucial role in disease progression and therapeutic response. Probiotics demonstrate therapeutic potential in IBD management by modulating intestinal microbiota, regulating histamine levels, and enhancing vitamin D metabolism, thereby promoting tolerogenic immune states and reducing inflammation (202). FMT, as a direct method to alter recipient microbial composition, has shown promise in IBD management across multiple studies. FMT may exert beneficial effects on IBD through modulating immune responses, restoring mucosal barrier integrity, and altering microbial metabolites (203). However, FMT application in IBD still faces challenges, including variability in donor selection criteria, standardization of transplantation protocols, and uncertainty regarding long-term post-transplantation outcomes (204). Furthermore, the quality of the clinical evidence for these adjunctive therapies remains inconsistent, with incompletely elucidated mechanisms of action and a lack of individualized application guidelines for different IBD subtypes, limiting their widespread clinical application and efficacy prediction.

The persistent limitations of current IBD therapies stem from their failure to comprehensively target the core pathogenic mechanisms underlying the disease. At the molecular level, existing treatments primarily target downstream inflammatory effector molecules, while failing to address upstream immune homeostatic imbalances. Although biologics, such as anti-TNF agents, can block specific inflammatory pathways, they exhibit limited ability to restore the Th17/Treg cell balance or repair innate immune defects, leaving the fundamental drivers of inflammatory responses intact (205). Most importantly, conventional therapies neglect the systemic dysfunction of the gut microecological-host immune network. Gut dysbiosis, disrupted short-chain fatty acid metabolism, and breakdown of the microbial-epithelial barrier-immune cell multivariate interaction network constitute the microenvironmental foundation for disease chronicity and recurrence; however, current pharmaceuticals lack effective interventions targeting these mechanisms. At the tissue repair level, current therapies exhibit pronounced mechanistic deficiencies in intestinal barrier reconstruction. Tight junction protein dysfunction, impaired mucus layer remodeling, and disrupted epithelial regeneration programs result in loss of barrier integrity, whereby continuous antigen exposure can trigger immune response reactivation even when inflammation is controlled, accounting for the fundamental inability to maintain sustained mucosal healing. Furthermore, a fundamental contradiction exists between the molecular endotype heterogeneity of IBD and current therapeutic strategies. Current evidence suggests that individual differences in genetic background, epigenetic modifications, microbiome characteristics, and immune phenotypes determine response patterns to specific treatments, while the absence of precision stratification strategies inevitably leads to therapeutic uncertainty (206).

Based on these considerations, the past few years have witnessed an increasing number of studies on emerging therapies. Research targeting various components of the gut microenvironment, including gut microbiota, intestinal metabolites, and immune pathways, has demonstrated promising potential in improving IBD outcomes (**Table 1**). In this regard, butyrate has been shown to alleviate intestinal inflammation by activating HIF-1 and promoting the expression of tight junction proteins. Additionally, acetate has been shown to yield protective effects on the intestinal barrier in organoid models derived from UC patients. Similarly, interventions targeting immune pathways, such as modulation of Th17 or Treg responses, are under active investigation.

**Table 1:** Therapeutic targeting multicomponents of the intestinal microenvironment.

Intestinal microenvironment	Components	Research target	Research subject	Research content (references)
Gut microorganisms	Bacteria	Beneficial gut bacteria	*VSL<ns/>3*	VSL<ns/>3, a mixture of 8 probiotic bacteria, has been confirmed to alleviate DSS-induced colitis by downregulating Tfh cells (125).
			*Bifidobacterium*	Bifidobacterium breve alters immune function and ameliorates DSS-induced inflammation in rats (126).
				Bifidobacterium longum CCM 7952 promotes epithelial barrier function and prevents acute DSS-induced colitis (127).
			*Lactobacillus*	Oral administration of Lactobacillus plantarum K68 ameliorates DSS-induced ulcerative colitis in BALB/c mice via the anti-inflammatory and immunomodulatory activities (128).
				Lactobacillus casei prevents the development of dextran sulphate sodium-induced colitis in Toll-like receptor 4 mutant mice (129).
				Lactobacillus reuteri prevents colitis by reducing P-selectin-associated leukocyte- and platelet-endothelial cell interactions (130).
				Lactobacillus rhamnosus alleviates intestinal barrier dysfunction in part by increasing expression of zonula occludens-1 and myosin light-chain kinase *in vivo* (131).
		Pathogenic bacteria	*Enterococcus faecalis*	Enterococcus faecalis EF-2001 attenuated IBD symptoms, suppressing the pathogenic shortening of colon length, reducing mesenteric lymph node weight, and downregulating proinflammatory cytokine expression in the colon, thereby improving Dinitrobenzene sulfonic acid induced colonic tissue destruction (132).
			*Enterococcus durans*	Enterococcus durans TN-3 induces regulatory T cells and suppresses the development of dextran sulfate sodium (DSS)-induced experimental colitis (133).
	Fungi	Fungi	*Saccharomyces boulardii*	Oral gavage of *Saccharomyces boulardii* supernatant (SbS) alleviated gut inflammation, protected the intestinal barrier, and reversed DSS-induced down-regulated activation of epidermal growth factor receptor (EGFR) in colitis (134).
	Viruses	Phage	Phage consortia	Targeted suppression of human IBD-associated gut microbiota commensals by phage consortia for treatment of intestinal inflammation (135).
Gut metabolites	Short-chain fatty acids	Butyrate	Butyrate	Butyrate attenuates intestinal inflammation and improves intestinal barrier by activation of HIF-1 (136).
			N-butyrate	N-butyrate upregulates intestinal claudin-23 expression and enhances barrier function through sp1 and ampk pathways in mouse colon and human intestinal caco-2 cells (137).
		Acetate	High acetate	High acetate protects intestinal barrier, reduces inflammation, and upregulates barrier genes in UC patient-derived organoids (138).
	Secondary Bile Acid	Secondary Bile Acid	Lithocholic acid (LCA)	Oral LCA suppresses inflammatory cytokines IL-6, IL-17α, and TNF-α to reduce DSS-induced colitis in mice in a VDR-dependent way. Concurrently, LCA treatment shows an increase in claudin-15 levels (139).
			Ursodeoxycholic acid (UDCA)/ Tauroursodeoxycholic acid (TUDCA)/ Glycoursodeoxycholic acid (GUDCA)	Giving ursodeoxycholic acid (UDCA), tauroursodeoxycholic acid (TUDCA), or glycoursodeoxycholic acid (GUDCA) orally every day can lessen the impact of DSS-induced colitis in mice (140).
	Tryptophan metabolite	Indole	Indoleacrylic Acid (IA)	IA significantly reduced the production of pro-inflammatory mediators, including PGE2, TNF-a, IL-6, and IL-8, while upregulating MUC2, AhR, and tight junction proteins, thereby enhancing mucosal barrier integrity (141).
Intestinal barrier	Epithelial cells layer	Tight Junctions	Larazotide acetate	Larazotide acetate, a zonulin antagonist, has been shown to prevent the disassembly of TJs by competitively inhibiting the binding of zonulin to its receptor (142).
		Intestinal Epithelial Cells	Intestinal stem cell /Goblet cells	Colonic epithelial-derived FGF1 drives intestinal stem cell commitment toward goblet cells to suppress inflammatory bowel disease (143).
			Lgr5+stem cells	Notoginsenoside R1 alleviates DSS-induced colitis in mice by promoting the regeneration of Lgr5+stem cells and intestinal reconstruction (144).
	Mucus Layer	Mucus barrier	Mucus barrier	Many herbs exhibit excellent anti-inflammatory effects, and have been shown to treat IBD by restoring the integrity of the mucus barrier (145).
	Antimicrobial Peptides	Endogenous Antimicrobial Peptides	α-defensin	Aryl hydrocarbon receptor-mediated induction of alpha- defensin 1 in colitis mice reversed the gut microbial dysbiosis and alleviated colitis (146).
			β-defensin	Engineered lactococcus lactis expressing mouse β-defensin 14 (L. lactis/mBD14) significantly alleviated DSS-induced colitis (147).
Intestinal immune system	Innate immune response	Pattern Recognition Receptors	Toll-like receptor	The protective effect of amitriptyline on experimental colitis through inhibiting Toll-like receptor 4/myeloid differentiation factor 2 signaling pathway (148).
			NOD-like receptor	Deubiquitinase USP14 is upregulated in Crohn's disease and inhibits the nucleotide binding oligomerization domain containing 2 pathway mediated inflammatory response in vitro (149).
		Macrophages	Macrophages polarization	The mesalamine prodrug nanoassemblies target pro-inflammatory macrophages in the intestinal tract through mucoadhesive properties and cathepsin B-triggered release of 5-ASA, promoting M2 macrophage polarization and epithelial cell repair (150).
				LMT503 is a therapeutic candidate that can target macrophages to drive polarization with an immunosuppressive character and ameliorate IBD (151).
				Tofacitinib Affects M1-like and M2-like Polarization and Tissue Factor Expression in Macrophages of Healthy Donors and IBD Patients (152).
		Neutrophil	Neutrophil extracellular traps(NET)	Efforts to pharmacologically manipulate NETs have resulted in the development of experimental treatments aimed at impeding NET formation or improving its elimination (153).
		Tolerogenic Dendritic Cell	Tolerogenic Dendritic Cell (tolDC)	Ex vivo generation of tolDCs via exposure to a suitable antigen could represent a potential therapeutic tool to re-induce tolerance and ameliorate inflammation in a number of conditions (154).
	Adaptive immune response	T cell	Target T cell	Several drugs have developed to target IBD pathogenesis via surface receptors, via T cell derived cytokines, via CRAC channels on T cells or by inducing T cell apoptosis (155).
			IL-12/23 antagonist	Targeting IL-23 was associated with significant therapeutic benefits in clinical trials involving patients with IBD and has led to the approval of ustekinumab for the treatment of both CD and UC, and risankizumab for the treatment of moderate-to-severe CD (156).
			Th17	Currently, the main targeted therapy methods include inhibiting the differentiation and proliferation of Th17 cells, neutralizing or inhibiting cytokines produced by Th17 cells, inhibiting the migration of Th17 cells (157).
			Treg/Th2	B.adolescentis ameliorates chronic colitis by regulating Treg/Th2 response and gut microbiota remodeling (158).
			Th1/Th17	Qingchang suppository ameliorates mucosal inflammation in ulcerative colitis by inhibiting the differentiation and effector functions of Th1 and Th17 cells (159).
		B cell	B-cell activating factor (BAFF)	B-cell activating factor (BAFF) expression is associated with Crohn's disease and can serve as a potential prognostic indicator of disease response to Infliximab treatment (160).

Clinical research data further substantiate the efficacy of these emerging therapies. In the realm of biologics, the selective interleukin-23 antagonist mirikizumab demonstrated significant efficacy in the phase III randomized controlled trials LUCENT-1 and LUCENT-2, with a clinical remission rate of 49.9%, compared to 25.1% in the placebo group (*P* < 0.001), thereby confirming the effectiveness of this novel targeted therapy (207). Similarly, in the domain of small molecule drugs, the JAK inhibitor upadacitinib showed superior induction of remission in phase 2b trials relative to placebo, offering a new oral treatment option, particularly for patients with inadequate responses to traditional biologics (208). In the field of innovative nanoparticle therapies, oral creatine-modified selenium hyaluronic acid nanogels significantly improved the Disease Activity Index (DAI), reducing the score from 4.2 to 1.8 in a DSS-induced colitis model. This improvement was accompanied by a 3.2-fold increase in ZO-1 expression and a 2.7-fold increase in occludin expression, demonstrating remarkable intestinal barrier repair capabilities (197). Furthermore, probiotic nanocomposites, through their excellent anti-inflammatory targeting and multiple therapeutic effects, significantly enhanced colitis treatment, reducing colonic inflammation scores by 75%, improving intestinal permeability, and significantly decreasing the pro-inflammatory cytokines IL-6 and TNF-α, thereby offering a novel technological platform for personalized treatment strategies (193).

While these emerging therapies have shown promise, their integration into clinical practice requires overcoming significant challenges, including variability in patient responses and the complexity of interactions between the gut microbiota and the host. As research progresses, these therapies have the potential to complement existing pharmacological and surgical interventions, offering a more comprehensive approach to improving patient outcomes.

In summary, while existing treatments have improved the quality of life for patients with IBD to some extent, there remains a pressing need for new therapeutic strategies. Addressing the shortcomings and limitations of current therapies is essential to enhance treatment efficacy, minimize side effects and achieve better long-term outcomes for patients. Research into innovative therapies and a deeper understanding of disease mechanisms will be vital for advancing IBD management and ultimately improving patient care.

## 6 The gut microenvironment in IBD: implications and future directions

The dysregulated interplay among the gut microbiota, microbial metabolites, impaired barrier integrity, and the host immune response forms a core pathological mechanism underlying the development of IBD. This positive feedback loop drives chronic inflammation through multi-level interactions, exacerbating repeated damage to intestinal tissues and ultimately triggering severe pathological changes, such as chronic ulcer formation, fibrosis, and dysfunction.

Dysbiosis often initiates this process, characterized by a reduction in beneficial bacteria and an overgrowth of pathogenic bacteria, leading to an imbalance of key metabolites. Indeed, this imbalance disrupts the intestinal physical and chemical barriers, resulting in the leakage of harmful microbial products into the lamina propria and the activation of both the innate and adaptive immune systems. Various immune cells are now understood to be sequentially activated and recruited in response to signaling, performing their functions and releasing pro-inflammatory factors and ROS, further exacerbating the inflammatory state. This, in turn, leads to intestinal barrier damage and aggravates dysbiosis, perpetuating a vicious loop of inflammation. Ultimately, this positive feedback mechanism not only results in a chronic inflammatory state of IBD but also facilitates lesion expansion and disease progression.

This complex pathological mechanism suggests that IBD is not driven by a single factor, but instead results from the interplay of microbiota, metabolism, barrier integrity, and immunity. Breaking this loop requires an integrated approach that combines strategies such as microbiota modulation, barrier repair, and immune regulation to effectively alleviate inflammation, halt disease progression, and ultimately restore intestinal homeostasis and health.

Future IBD treatment needs to shift focus from “controlling inflammation” to “restoring intestinal homeostasis,” and from “single therapy” to “comprehensive intervention”. Given the pivotal role of the gut microenvironment in the pathogenesis of IBD, we propose that future therapeutic directions should focus on regulating the multidimensional microbiome. An integrated approach may include gut microbiota modulation, metabolite supplementation (e.g., butyrate and indole metabolites), immune microenvironment reconstruction (including regulation of the Th17/Treg balance and the use of inflammasome inhibitors), barrier repair techniques (e.g., TJ protein regulators and ISC therapy), and the incorporation of gene editing technologies. Moreover, integrating multi-omics techniques, such as metabolomics, transcriptomics, and immunomics, can provide a deeper understanding of the gut microenvironment in IBD. Importantly, the introduction of nanoparticle delivery systems offers innovative strategies to disrupt the deleterious feedback loop in IBD, enabling the precise delivery of therapeutic agents (e.g., anti-inflammatory cytokines, probiotics, or compounds that repair the intestinal barrier) directly to the inflammatory sites. This approach minimizes systemic side effects and enhances treatment efficacy. Recent advances have led to the development of a diverse array of nanomaterial-based drug delivery systems (NDDS), encompassing inorganic frameworks, organic polymers, bio-nanomaterials, and engineered microbe-based platforms. Each class exhibits unique physicochemical properties, such as pH-responsiveness, enzymatic sensitivity, or ligand targeting, that are exploited for site-specific delivery, redox regulation, immune modulation, or mucosal repair in IBD. **Table 2** provides a comprehensive classification of these NDDS along with representative studies that exemplify their therapeutic potential. These nanoparticle delivery platforms can accurately control the drug release rate according to clinical needs, allowing for the sequential delivery of multiple drugs as required, which can promote synergistic effects of medications. Thus, facilitating personalized, high-efficacy interventions. This strategy aims to enhance therapeutic effects and prolong efficacy. Such multidimensional combined strategies, through their synergistic effects, have the potential to achieve more efficient and lasting clinical remission and restore intestinal homeostasis, representing a promising new direction for future IBD treatment.

**Table 2:** NDDS in inflammatory bowel disease: classification and representative studies.

NDDS	Nanoparticles (NPs)	Category	Properties	Representative studies
Inorganic NDDS	Metals	Metal-organic framework	Metal-organic frameworks with tunable porosity for pH-responsive drug release and ROS scavenging.	Biomimetic MOF Nanoparticles Delivery of C-Dot Nanozyme and CRISPR/Cas9 System for Site-Specific Treatment of Ulcerative Colitis (161).
		Noble metal	Noble metal nanoparticles (Au, Ag, Pt) not only load drugs but also exhibit antioxidant and anti-inflammatory properties.	AuNPs effectively targeted the colonic tissue, and reduced changes induced by DSS. The underlying mechanisms could be related to anti-oxidant effect (as evident by decreasing tissue MDA) and anti-inflammatory potential of AuNPs (162).
		Metal oxide	Metal oxide nanoparticles (ZnO, Fe3O4) offer high drug loading and, in some cases, magnetic targeting for improved delivery.	ZnO NPs significantly improves TNBS-induced intestinal inflammatory damage by modulating the gut microbiota, mucus, and mechanical barriers (163).
	Non-metals	Mesoporous silica	Mesoporous silica nanoparticles with tunable pore sizes and pH-sensitive drug release for targeted colon delivery.	Given the special shapes of mesoporous silica nanoparticles (MSNs) and pH-responsivity of Eudragit S100, budesonide (BUD) loaded in the voids of MSND (E@MSNs-BUD) could penetrate the mucous layer and be accurately delivered to the colon with minor side effects (164).
		Carbon-based	Carbon-based nanomaterials (graphene, carbon dots) efficiently load drugs and offer biocompatibility, utilizing π-π stacking for drug adsorption.	Carbon dots based on Bletilla striata (BS-CDs) significantly increased colon length, improved colonic histopathology, and reduced the levels of pro-inflammatory cytokines (TNF-a, IL-1β, and IL-6) in colitis mice (165).
	Nanoenzyme	Cerium-based	Cerium-based nanozymes mimic SOD and CAT activities, effectively scavenging O2- and H2O2.	CeNP-PEG ameliorated the proinflammatory microenvironment by persistently scavenging ROS, down-regulating the levels of multiple proinflammatory cytokines, restraining the proinflammatory profile of macrophages and Th1/Th17 response (166).
		Selenium-based	Selenium-based nanozymes, with GPx-like activity, scavenge peroxides and restore redox balance.	Zero-Valence Selenium-Enriched Prussian Blue Nanozymes Reconstruct Intestinal Barrier against Inflammatory Bowel Disease via Inhibiting Ferroptosis and T Cells Differentiation (167).
		Nickel-based	Nickel-based nanozymes (e.g., Ni3S4) with multiple enzymatic activities to scavenge ROS/RNS.	As demonstrated in a mouse model, Ni3 S4 is stable in the gastrointestinal tract without toxicity and specifically targets the diseased colon to alleviate oxidative stress. RNA and 16S rRNA sequencing analyses show that Ni3 S4 effectively inhibits the cellular pathways of pro-inflammatory factors and restores the gut microbiota (168).
		Platinum-based	Platinum-based nanozymes mimic SOD and CAT activities, scavenging O2- and H2O2.	Platinum nanoparticles (Pt NPs) exhibited remarkable superoxide dismutase (SOD) and catalase (CAT) cascade catalytic activities, as well as effective hydroxyl radical (•OH) scavenging ability. The *in vitro* experiments showed that Pt NPs could eliminate excessive ROS to protect cells against oxidative stress (169).
Organic NDDS	Lipid	Conventional liposomes	Liposomes, encapsulate both hydrophilic and hydrophobic drugs, improving stability and bioavailability.	Krill Oil-Incorporated Liposomes As An Effective Nanovehicle To Ameliorate The Inflammatory Responses Of DSS-Induced Colitis (170).
		Solid lipid nanoparticles (SLN)	SLNs, made from solid lipids, provide improved stability and sustained release properties.	Improved uptake and therapeutic intervention of curcumin via designing binary lipid nanoparticulate formulation for oral delivery in inflammatory bowel disorder (171).
		Nanostructured lipid carriers (NLC)	NLCs, composed of solid-liquid lipids, overcome SLN drawbacks with improved drug encapsulation.	Oral delivery of oleuropein-loaded lipid nanocarriers alleviates inflammation and oxidative stress in acute colitis (172).
		Lipid-like nanoparticles	Lipid-like nanoparticles, chemically modified to mimic natural lipids, offer high biocompatibility and targeted drug delivery.	Oral delivery of IL-22 mRNA-loaded lipid nanoparticles targeting the injured intestinal mucosa: A novel therapeutic solution to treat ulcerative colitis (173).
	Polysaccharide	Chitosan-based	Chitosan nanoparticles enhance drug release and absorption with anti-inflammatory and immune-modulatory effects.	Melatonin-loaded chitosan nanoparticles endows nitric oxide synthase 2 mediated anti-inflammatory activity in inflammatory bowel disease model (174).
		Hyaluronic acid-based	Hyaluronic acid-based nanoparticles target CD44 receptors in inflamed tissues for localized drug delivery.	Budesonide-Loaded Hyaluronic Acid Nanoparticles for Targeted Delivery to the Inflamed Intestinal Mucosa in a Rodent Model of Colitis (175).
		Alginate-based	Alginate-based nanoparticles, stable in acidic conditions, degrade in the colon for colon-specific drug release.	SNase encapsulated with calcium alginate (ALG-SNase) was formulated using crosslinking technology with sodium alginate and calcium chloride. Oral administration of ALG-SNase nanoparticles decreased NET levels in the colon and effectively alleviated the clinical colitis index and tissue inflammation in UC mice (176).
		Pectin-based	Pectin-based nanoparticles degrade in the colon, enabling targeted drug release.	Pectin-coated polymeric NPs encapsulating the inflammation-resolving peptide Ac2–26 reduced colitis activity postoperatively (177).
		Dietary polysaccharide-based	Dietary polysaccharide-based nanoparticles modulate immune function, enhance gut barrier, and restore microbiota to alleviate IBD.	Polysaccharides from tragacanth gum (GUM) are coordinated with iron and further interact with shikonin (Shik) to obtain a nanocomposite named Shik-Fe@GUM, which can modulate IL-17RA relevant cell signaling and reduce the recruitment of Th17 cells (T helper cell 17) (178).
		Chondroitin sulfate-based	Chondroitin sulfate-based nanoparticles target CD44 receptors on inflammatory cells, offering bioadhesive properties.	The mechanism of Ta2 C Modified with Chondroitin Sulfate (TACS) treatment mainly involves protection of mitochondria, elimination of oxidative stress, inhibiting macrophage M1 polarization, protection of intestinal barrier, and restoration of intestinal flora balance (179).
	Protein	Silk fibroin	Silk fibroin, a biocompatible protein, protects drugs from gastric degradation and enhances stability.	Silk fibroin nanoparticles enhance quercetin immunomodulatory properties in DSS-induced mouse colitis (180).
		Albumin	Albumin, with high biocompatibility, targets inflammation via SPARC in inflamed tissues.	Heparin-Coated Albumin Nanoparticles for Drug Combination in Targeting Inflamed Intestine (181).
		Casein	Casein offers high biocompatibility and gastric stability.	GCPP NPs was synthesized through covalent assembly of Genipin and Casein phosphopeptide (CPP), which can passively accumulate and maintain at inflamed sites. The body weight and colon length of DSS-induced colitis mice treated by GCPP NPs perform a rehabilitation trend (182).
		Gelatin	Gelatin, a collagen hydrolysate, is biodegradable by proteases for colon-specific release.	A multicompartmental biodegradable polymer-based nanoparticles-in-microsphere oral system (NiMOS) using gelatin nanoparticles encapsulating a combination of siRNA duplexes specifically targeted against tumor necrosis factor-α (TNF-α) and cyclin D1 (Ccnd1) was employed to study its effects on a dextran sulfate sodium (DSS)-induced acute colitis mouse model mimicking inflammatory bowel disease (IBD) (183).
	Polymer-Based	Poly (lactic-co-glycolic acid) (PLGA)	PLGA, a biodegradable polymer, offers biocompatibility and controlled release.	Dual action tofacitinib-loaded Poly(lactic-co-glycolic acid) (PLGA) nanoparticles alleviate colitis in an IBD mouse model (184).
		Polyacrylic acid	Polyacrylic acid, a pH-sensitive polymer, releases drugs in the alkaline conditions of the colon.	Budesonide-Loaded Eudragit S 100 Nanocapsules for the Treatment of Acetic Acid-Induced Colitis in Animal Model (185).
		Polyethylenimine	Polyethylenimine, a cationic polymer, is ideal for gene delivery due to high transfection efficiency.	Functional TNF-a gene silencing mediated by polyethyleneimine/TNF-a siRNA nanocomplexes in inflamed colon (186).
		PEGylated nanoparticles	PEGylated nanoparticles enhance stability, circulation time, and reduce immune clearance.	Optimizing PLGA-PEG Nanoparticle Size and Distribution for Enhanced Drug Targeting to the Inflamed Intestinal Barrier (187).
	Bio-nanomaterial-related	Cell membrane-derived	Cell membrane-derived nanoparticles, wrapped in red or white blood cell membranes, provide immune evasion and targeted delivery.	A ROS scavenging and inflammation-directed nanomedicine is designed and fabricated by coupling polydopamine nanoparticles with mCRAMP, an antimicrobial peptide, while wrapping macrophage membrane in the outer layer (188).
		Exosome-based	Exosome-based nanoparticles offer natural cell targeting and membrane penetration abilities.	Cerium Oxide-Loaded Exosomes Derived From Regulatory T Cells Ameliorate Inflammatory Bowel Disease by Scavenging Reactive Oxygen Species and Modulating the Inflammatory Response (189).
		Plant-derived	Plant-derived nanoparticles provide high biocompatibility and specific targeting.	Amelioration of colitis progression by ginseng-derived exosome-like nanoparticles through suppression of inflammatory cytokines (190).
		Bacterial-derived	Bacterial-derived nanoparticles target the gut with high precision.	Odoribacter splanchnicus-derived extracellular vesicles alleviate inflammatory bowel disease by modulating gastrointestinal inflammation and intestinal barrier function via the NLRP3 inflammasome suppression (191).
	Engineered Microbe-Based	Engineered probiotic	Engineered probiotics deliver therapeutic factors or nanoparticles, colonizing the gut for sustained effects.	Engineered Probiotics Enable Targeted Gut Delivery of Dual Gasotransmitters for Inflammatory Bowel Disease Therapy (192).
		Bacteria-nanomaterial hybrid systems	Bacteria-nanomaterial hybrid systems combine bacterial motility with nanomaterial-based drug delivery.	The author present an innovative therapeutic strategy for encapsulating probiotic Bacillus coagulans spores with rosmarinic acid (RA) and silk fibroin (SF) (193).
		Phage display systems	Phage display systems target gut pathogens or inflammatory cells with high specificity.	Designing and constructing a phage display synthesized single domain antibodies library based on camel VHHs frame for screening and identifying humanized TNF-a-specific nanobody (194).
Composite NDDS	Inorganic-organic hybrid nanocarriers	Metal-polysaccharide composite nanomaterials	Metal-polysaccharide composite nanomaterials combine metal nanoparticle stability with polysaccharide biocompatibility.	Iron oxide particles are embedded into Chitosan Milliwheels for Rapid Translation, Barrier Function Rescue, and Delivery of Therapeutic Proteins to the Inflamed Gut Epithelium (195).
		Inorganic nanomaterial-liposome composite systems	Inorganic nanomaterial-liposome composites combine functional inorganic nanoparticles with liposome biocompatibility.	CeO2@S100 is composed of a CeO2 nanoparticle core and a protective polyacrylic acid resin shell (Eudragit S100),regulating the redox balance and gut microbiome (196).
	Multi-component Functionalized Nanocarriers	Targeted ligand-modified nanocarriers	Ligand-modified nanocarriers enhance targeting through specific surface modifications.	An oral creatine-modified selenium-based hyaluronic acid (HA) nanogel (HASe-Cr nanogel) was fabricated for treatment of IBD, through ROS elimination and energy metabolism upgradation (197).
		Multistimuli-responsive nanocarriers	Multistimuli-responsive nanocarriers enable precise drug release based on environmental triggers.	Enhanced therapeutic efficacy of budesonide in experimental colitis with enzyme/pH dual-sensitive polymeric nanoparticles (198).

## 7 Conclusions

The systemic dysregulation of the gut microenvironment represents a fundamental pathophysiological mechanism driving IBD pathogenesis through complex interactions among microbial dysbiosis, alterations in metabolite levels, barrier dysfunction, and immune activation. This review has elucidated how the disruption of beneficial microbiota and the proliferation of pathogenic organisms initiate a cascade that compromises intestinal homeostasis. Reduced protective metabolites and impaired tight junction integrity facilitate microbial translocation and sustained immune responses, perpetuating chronic inflammation. Current therapeutic approaches, while providing symptomatic relief, remain inadequate for achieving long-term disease control due to their failure to address the multifaceted nature of gut microenvironmental dysregulation. The limitations of conventional pharmacotherapy, surgical intervention, and adjunctive therapies underscore the urgent need for treatment strategies that evolve from “controlling inflammation” to “restoring intestinal homeostasis” through comprehensive, multidimensional interventions. Future IBD management should simultaneously target microbiome remodeling, metabolite supplementation, immune microenvironment reconstruction, and barrier repair through integrated approaches incorporating multi-omics technologies, gene editing, and nanoparticle delivery systems. These innovative platforms offer unprecedented opportunities for precision medicine by enabling targeted therapeutic delivery, minimizing systemic toxicity, and facilitating synergistic treatment effects. By understanding and therapeutically targeting the intricate network of gut microenvironmental interactions, we can disrupt the pathological feedback loops that sustain chronic inflammation and work toward achieving durable remission and restoration of intestinal homeostasis in patients with IBD.
